# Asymptomatic Intracardiac Migration of a Cane-Shaped Polymethylmethacrylate Cement Embolus Following Percutaneous Vertebroplasty

**DOI:** 10.14740/jmc5403

**Published:** 2026-10-02

**Authors:** Jinchul Noh, Sanghoun Lee, Youngmin Oh, Jongpil Eun

**Affiliations:** a Department of Neurosurgery, Research Institute of Clinical Medicine, Biomedical Research Institute, Jeonbuk National University Medical School and Hospital, Jeonju 54907, Korea

**Keywords:** Percutaneous vertebroplasty, Polymethylmethacrylate, Bone cement embolism, Intracardiac embolism

## Abstract

Percutaneous vertebroplasty (PVP) is a minimally invasive and effective treatment for osteoporotic vertebral compression fractures. Although generally safe, cement leakage is a relatively common complication and may rarely result in intracardiac embolism. We report the case of a 75-year-old woman who underwent PVP for an L2 compression fracture after failed conservative treatment. Postoperative imaging revealed venous cement leakage, but she remained asymptomatic. Six months later, during a routine health examination, echocardiography incidentally detected an abnormal structure in the right atrium. Cardiac computed tomography demonstrated a linear, cane-shaped polymethylmethacrylate embolus extending from the right atrium to the right ventricle. Despite the absence of cardiopulmonary symptoms, surgical removal was performed because of the elongated and sharp morphology of the embolus and the associated risk of cardiac perforation. Open-heart surgery was successfully completed without major complications. Although many intracardiac cement embolisms are asymptomatic and can be managed conservatively, embolus morphology and location should guide treatment decisions. Early recognition and individualized management are essential to prevent life-threatening complications.

## Introduction

Percutaneous vertebroplasty (PVP) was developed in 1987 by Galibert and Deramond in France and has been used for various spinal disorders (osteoporotic vertebral fractures, traumatic fractures, benign tumors, and malignant tumors) over the past four decades [1]. Various types of bone cements have been developed, but polymethylmethacrylate (PMMA) remains the most commonly utilized in PVP [2].

PVP provides mechanical stabilization of the vertebral body and reduces pain. The rate of pain reduction has been reported to be between about 70% and 100% [3, 4].

PVP is considered a relatively safe and simple procedure owing to its minimally invasive nature; however, complications occur in approximately 1–10% of cases [5]. These complications are mostly asymptomatic but can be classified into three main categories based on their severity: mild, moderate, and severe [6, 7].

The main cause of these complications is cement leakage, reported to occur in about 30–65% of cases [8]. Among these, venous leakage is the most concerning, as it can lead to pulmonary and cardiac problems owing to venous cement embolism. We report a case of an incidentally discovered intracardiac PMMA embolism approximately 6 months after PVP, in which the patient remained asymptomatic.

## Case Report

A 75-year-old female presented to our outpatient clinic with lower back pain. She had a history of osteoporosis at another hospital and had previously undergone vertebroplasty for a compression fracture at L1. Lumbar spine magnetic resonance imaging revealed a new compression fracture at L2 (Fig. 1).

**Figure 1:**
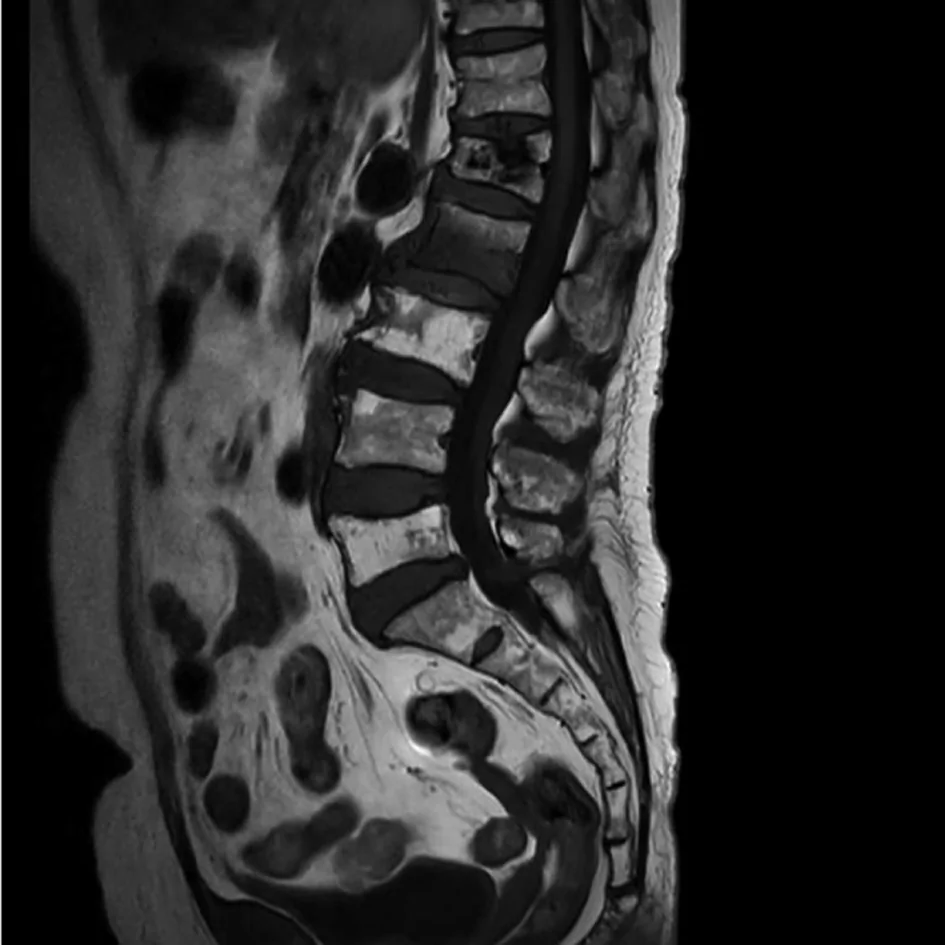
Sagittal lumbar spine MRI. The T1-weighted image demonstrates a new acute compression fracture at the L2 vertebral body. MRI: magnetic resonance imaging.

After 2 weeks of conservative treatment, the patient continued to complain of persistent lower back pain; therefore, PVP was performed. Under local anesthesia in the prone position, a Jamshidi needle was used, and PVP was performed on the right side of L2 using a transpedicular approach. PMMA (3 mL) was then injected. Postoperative radiography revealed venous leakage of the bone cement (Fig. 2a, b). The patient remained asymptomatic without cardiopulmonary symptoms, and no additional imaging was performed to evaluate possible cement migration at that time. Her back pain improved significantly and she was discharged 2 days later.

**Figure 2:**
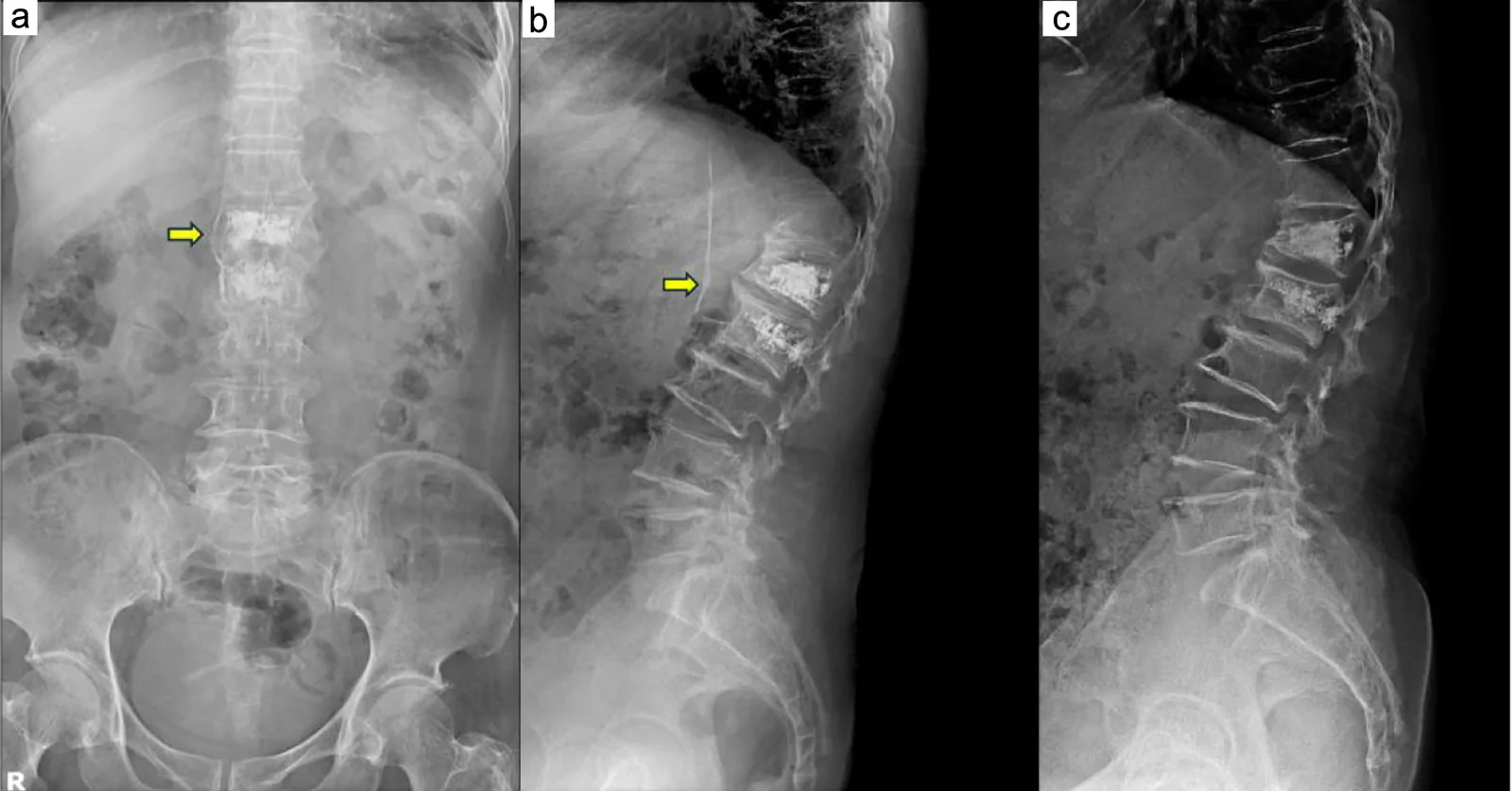
Postoperative and follow-up lumbar radiographs. (a, b) Postoperative anteroposterior and lateral radiographs showing venous leakage of the bone cement following percutaneous vertebroplasty at L2. (c) Follow-up lumbar radiograph obtained 2 weeks later, showing that the previously observed venous cement leakage was no longer visible.

Two weeks after discharge, she returned to the outpatient clinic for follow-up. A lumbar radiograph showed that the previously observed venous cement leakage was no longer visible (Fig. 2c). Because the patient remained asymptomatic, no additional imaging studies were performed at that time to evaluate possible migration of the cement into the inferior vena cava, right heart, or pulmonary circulation.

Six months later, during a routine health checkup at another hospital, echocardiography revealed abnormal findings in the right atrium. Cardiac computed tomography showed a linear foreign body (bone cement) extending across the right atrium (RA) and right ventricle (RV) (Fig. 3a, b).

**Figure 3:**
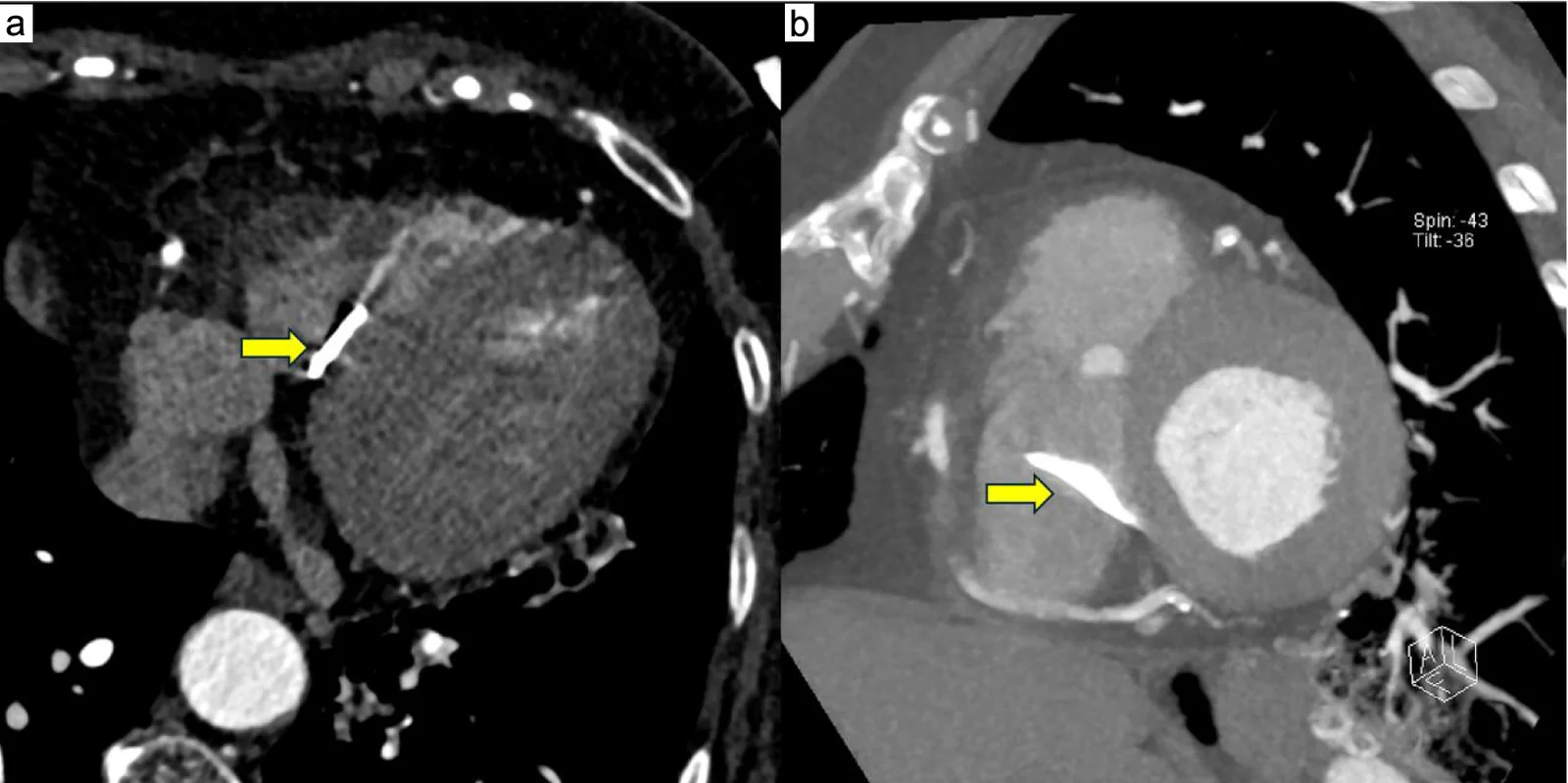
Cardiac CT scans. (a, b) Axial and reconstructed views showing a linear, cane-shaped hyperdense foreign body extending from the RA into the RV. CT: computed tomography; RA: right atrium; RV: right ventricle.

The embolus was cane-shaped and extended from the RA into the RV. Although the patient remained asymptomatic, the elongated and sharp morphology of the embolus and its extension into the right ventricle raised concern for cardiac perforation and valvular injury. Following multidisciplinary discussion involving an interventional cardiologist, a cardiac surgeon, and a neurosurgeon, surgical removal was favored because of the embolus morphology, location, and the potential risk of serious cardiac complications.

Open-heart surgery was performed for cement removal. After median sternotomy, heparinization, and pericardiotomy, bicaval cannulation and cardiopulmonary bypass were established, and systemic cooling was initiated. The cement extended into the septal leaflet and penetrated the RV side. Cane-shaped cement was removed with partial division of the septal leaflet (Fig. 4). Tricuspid valve ring annuloplasty and right atriotomy were performed. The procedure was completed without major complications, and the patient was discharged 1 week later.

**Figure 4:**
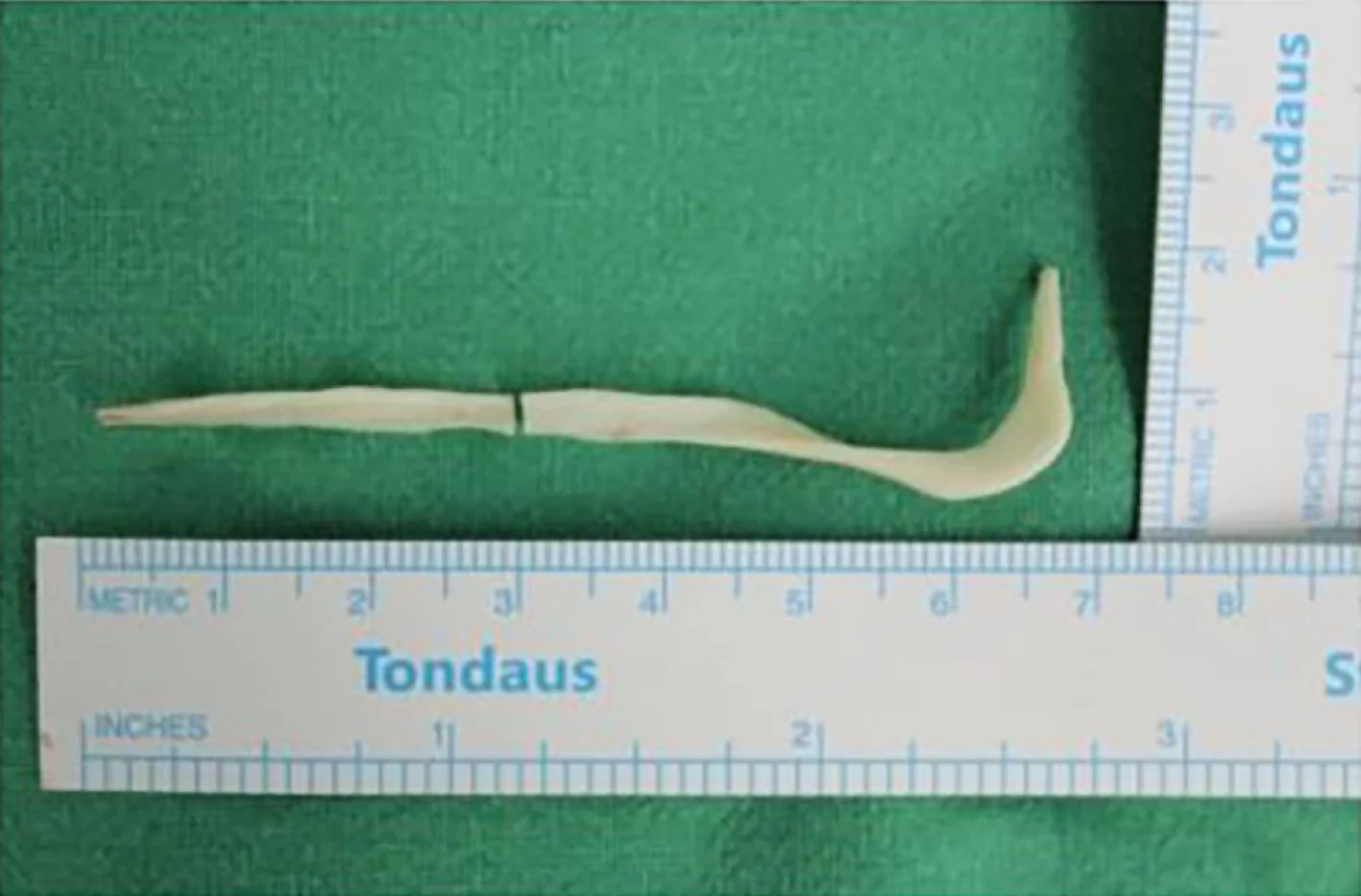
Intraoperative photograph of the removed bone cement. The extracted cane-shaped bone cement after successful surgical removal via open-heart surgery.

## Discussion

PVP is a safe and effective procedure for pain relief; however, complications owing to cement leakage may occur. The frequency of complications is reportedly 1–10% [5], with cement leakage occurring in 30–65% of cases [8]. Venous cement leakage occurs in about 24% [9]. Complications can be categorized as mild, moderate, or severe. Mild complications include transient hypotension and cement leakage into the intervertebral disc space or paravertebral soft tissue. Moderate complications include infection and cement leakage into the epidural or foraminal spaces. Severe complications include cement extravasation into the paravertebral vein, potentially leading to pulmonary and cardiac embolisms [6, 7].

Risk factors for cement leakage include intravertebral clefts, cortical disruption, low cement viscosity, and high injection volume [10]. In our case, no definite patient-specific risk factor for venous cement leakage could be identified retrospectively. To reduce the risk of venous cement leakage and subsequent embolization, careful fluoroscopic monitoring during cement injection, use of appropriately viscous cement, avoidance of excessive injection pressure or volume, and immediate cessation of injection when venous leakage is observed are important procedural considerations [6].

Intracardiac and pulmonary cement embolisms are of particular concern. Cement may migrate through the paravertebral veins into the azygos vein or inferior vena cava, then through the right atrium and ventricle, eventually reaching the pulmonary arteries [11]. In our case, migration from the L2 venous leakage to the right atrium and right ventricle was presumed based on the anatomical venous pathway but was not directly demonstrated on serial imaging. The incidence of intracardiac cement embolism is approximately 3.9% and it is usually asymptomatic [12]. The most common symptoms include chest pain and dyspnea; in severe cases, tamponade or perforation may occur [13, 14].

In a systematic review by Hatzantonis et al, conservative treatment was recommended for asymptomatic intracardiac cement embolisms, while surgical intervention was advised for symptomatic cases. Treatment may involve either open-heart surgery or percutaneous approaches, with generally favorable outcomes. Open surgical removal is particularly considered when the cement is located in the right ventricle because of the risk of perforation and other severe complications [15]. Conservative management in asymptomatic cases may include anticoagulation therapy, such as warfarin, until endothelialization reduces the thrombogenic risk [16].

When venous cement leakage is identified after vertebroplasty, further evaluation should be guided by the patient’s symptoms and the extent and morphology of the leakage. Additional imaging, such as chest radiography, computed tomography, or echocardiography, may be considered when migration into the cardiopulmonary circulation is suspected [12, 15]. In asymptomatic patients without evidence of high-risk intracardiac embolization, close clinical and imaging follow-up may be appropriate [15, 16]. In contrast, intervention should be considered when the embolus is intracardiac, elongated or sharp, associated with right ventricular involvement, or considered to carry a substantial risk of cardiac perforation or valvular injury. Accordingly, management should be individualized through multidisciplinary assessment based on symptoms, embolus morphology, location, and the potential risk of future complications [15]. Our case demonstrates that the absence of cardiopulmonary symptoms does not exclude delayed cement migration, emphasizing the need for continued clinical vigilance when venous cement leakage is identified.

However, several reports describe cardiac perforation caused by sharp or elongated cement embolisms [14, 17]. Intracardiac cement embolism may also present after a prolonged latent period and remain clinically silent until significant cardiac injury develops. Guo et al reported an ultra-delayed intracardiac cement embolism 2.5 years after vertebroplasty, with multiple rod-shaped cement fragments located in the right ventricle, including one embedded in the ventricular myocardium [18]. Setoguchi et al described a patient who remained asymptomatic for 6 months after vertebroplasty before presenting with cardiac tamponade due to right ventricular perforation by migrated bone cement [19]. A similar concern for valvular injury was illustrated by Hamade et al, who reported an intracardiac cement embolus causing extensive tricuspid valve damage that required surgical removal and valve replacement [20]. In our case, the patient remained asymptomatic for 6 months and the embolus was detected incidentally. Nevertheless, its long, sharp, cane-shaped morphology extending from the right atrium to the right ventricle raised concern for cardiac perforation or valvular injury. Intraoperative findings demonstrating involvement of the tricuspid septal leaflet and penetration toward the right ventricular side further supported the decision for surgical removal despite the absence of symptoms.

### Conclusion

PVP is generally a safe and effective procedure for pain relief. However, cement leakage can lead to serious complications, including intracardiac embolism. While many cases remain asymptomatic and may be managed conservatively, elongated and sharp emboli pose a risk of cardiac perforation. Treatment should be individualized based on embolus morphology, location, and patient symptoms. In our case, despite the absence of symptoms, surgical removal was performed owing to the high risk associated with the embolus shape and location.

## Data Availability

The data generated during this study are not publicly available because of patient privacy but are available from the corresponding author upon reasonable request.
